# Reasons for Receiving or Not Receiving Bivalent COVID-19 Booster Vaccinations Among Adults — United States, November 1–December 10, 2022

**DOI:** 10.15585/mmwr.mm7203a5

**Published:** 2023-01-20

**Authors:** Alyssa H. Sinclair, Morgan K. Taylor, Joshua S. Weitz, Stephen J. Beckett, Gregory R. Samanez-Larkin

**Affiliations:** ^1^Department of Psychology and Neuroscience, Duke University, Durham, North Carolina; ^2^School of Biological Sciences, Georgia Institute of Technology, Atlanta, Georgia; ^3^School of Physics, Georgia Institute of Technology, Atlanta, Georgia; ^4^Institut d’Biologie, École Normale Supérieure, Paris, France.

Bivalent COVID-19 booster vaccines, developed to protect against both ancestral and Omicron BA.4/BA.5 variants, are recommended to increase protection against SARS-CoV-2 infection and severe disease* (*1*,*2*). However, relatively few eligible U.S. adults have received a bivalent booster dose (*3*), and reasons for low coverage are unclear. An opt-in Internet survey of 1,200 COVID-19–vaccinated U.S. adults was conducted to assess reasons for receiving or not receiving a bivalent booster dose. Participants could select multiple reasons from a list of suggested reasons to report why they had or had not received a bivalent booster dose. The most common reasons cited for not receiving the bivalent booster dose were lack of awareness of eligibility for vaccination (23.2%) or of vaccine availability (19.3%), and perceived immunity against infection (18.9%). After viewing information about eligibility and availability, 67.8% of participants who had not received the bivalent booster dose indicated that they planned to do so; in a follow-up survey 1 month later, 28.6% of these participants reported having received the dose. Among those who had planned to receive the booster dose but had not yet done so, 82.6% still intended to do so. Participants who had still not received the booster dose most commonly reported being too busy to get vaccinated (35.6%). To help increase bivalent booster dose coverage, health care and public health professionals should use evidence-based strategies to convey information about booster vaccination recommendations and waning immunity (*4*), while also working to increase convenient access.

Participants were recruited via Prolific, an online survey platform.^†^ Eligible participants included persons who were aged ≥18 years, fluent in English, U.S. residents, and had received ≥1 previous COVID-19 vaccine dose. Quota-sampling was used to recruit approximately equal numbers of adults aged 18–39, 40–59, and ≥60 years. Because of low racial and ethnic diversity among persons in the Prolific participant pool (most identified as non-Hispanic White [White]), particularly among adults aged ≥60 years and those who had previously received a COVID-19 vaccine, the sample was not weighted by race or ethnicity. Data collection occurred during November 1–5, 2022 (initial survey), and December 6–10 (follow-up survey). CDC first recommended a bivalent booster dose for persons aged ≥12 years on September 1, 2022. Participants were not informed during the initial survey that they would later be recontacted for a follow-up survey. This study was reviewed and approved by the Duke University Institutional Review Board.^§^

Participants reported dates of all previous COVID-19 infections (as determined by positive rapid test results or reverse transcription-polymerase chain reaction test results) and COVID-19 vaccine doses. Participants who reported receiving a bivalent booster dose viewed a randomly ordered set of 10 suggested reasons for getting the booster dose,^¶^ and could select multiple reasons that contributed to their decision, as well as optionally input other reasons.** Similarly, participants who had not received a bivalent booster dose could select from a different randomly ordered set of 10 reasons for not getting the booster dose,^††^ and optionally input other reasons; they then viewed information about bivalent booster vaccine eligibility and availability.^§§^ After viewing this information, participants who had not received a bivalent booster dose reported whether they planned to receive it and were recontacted via Prolific after 1 month to complete a follow-up survey. Descriptive statistics were calculated using R (version 4.1.1; R Foundation). Survey materials, data, and code used for data preprocessing and analysis are available online.^¶¶^

The initial survey included 1,200 participants, with approximately one third in each age group (Table 1). Nearly two thirds (65.4%) of participants were White, and approximately one half (51.9%) were women. Most participants (95.8%) had received ≥2 COVID-19 vaccine doses; among these participants, 396 (34.4%) had received the bivalent booster dose, and 714 (62.1%) had not. Participants who had received only 1 vaccine dose (50) or were unsure if they had received a bivalent booster dose (41) were excluded from further analyses.

**TABLE 1:** Characteristics of participants in the initial survey,* by bivalent-booster dose status, and in the follow-up survey^†^ — United States, November 1–December 10, 2022

Characteristic	No. (%)			
	Initial survey participants			Follow-up survey participants (624)
	Total (1,200)	Received bivalent booster dose (396)	Did not receive bivalent booster dose (759)	
**Age group, yrs**				
18–39	**406 (33.8)**	101 (25.5)	287 (37.8)	225 (36.1)
40–59	**397 (33.1)**	120 (30.3)	262 (34.5)	225 (36.1)
≥60	**397 (33.1)**	175 (44.2)	210 (27.7)	174 (27.9)
**Race and ethnicity^§^**				
Asian	**132 (11.0)**	44 (11.1)	81 (10.7)	66 (10.6)
Black or African American	**103 (8.6)**	23 (5.8)	71 (9.4)	51 (8.2)
Hispanic or Latino	**89 (7.4)**	27 (6.8)	59 (7.8)	50 (8.0)
Alaska Native or Native American	**20 (1.7)**	5 (1.3)	13 (1.7)	11 (1.8)
White	**785 (65.4)**	271 (68.4)	491 (64.7)	409 (65.5)
Multiple races	**60 (5.0)**	20 (5.1)	40 (5.3)	34 (5.4)
Other	**11 (1.0)**	6 (1.5)	4 (0.5)	3 (0.5)
**Gender**				
Man	**559 (46.6)**	179 (45.2)	358 (47.2)	297 (47.6)
Woman	**623 (51.9)**	209 (52.8)	391 (51.5)	320 (51.3)
Other (nonbinary or prefer not to say)	**18 (1.5)**	8 (2.0)	10 (1.3)	7 (1.1)
**No. of COVID-19 vaccine doses received**				
1	**50 (4.2)^¶^**	0 (—)	45 (5.9)^¶^	0 (—)
2	**339 (28.5)**	23 (5.8)	307 (40.4)	264 (42.3)
3	**413 (34.4)**	69 (17.4)	328 (43.2)	288 (46.2)
4	**272 (22.7)**	185 (46.7)	75 (9.9)	68 (10.9)
5	**126 (10.5)**	119 (30.1)	4 (0.1)	4 (0.6)
**Bivalent booster dose received**				
Yes	**396 (33.0)**	396 (100.0)	0 (—)	131 (21.0)
No	**759 (63.3)**	0 (—)	759 (100.0)	493 (79.0)
Unsure	**41 (3.4)^¶^**	0 (—)	0 (—)	0 (—)
No. of previous SARS-CoV-2 infections**				
0	**680 (56.7)**	268 (67.7)	388 (51.1)	360 (57.7)
1	**363 (30.3)**	92 (23.2)	255 (33.6)	213 (34.1)
2	**80 (6.7)**	21 (5.3)	55 (7.2)	43 (6.9)
≥3	**12 (1.0)**	2 (0.1)	10 (1.3)	8 (1.3)

* The initial sample consisted of 1,200 previously COVID-19–vaccinated U.S. residents.

^†^ The follow-up survey consisted of 624 participants from the initial survey, recontacted after 1 month. Only participants who had reported not yet receiving the bivalent booster dose during the initial survey were recontacted.

^§^ Persons of Hispanic or Latino (Hispanic) origin might be of any race but are categorized as Hispanic; all racial groups are non-Hispanic. Persons identified as being of multiple races had more than one race category selected. Persons identified as “other” race selected the “other” option or identified as Native Hawaiian or other Pacific Islander.

^¶^ Participants who were unsure whether they had received a bivalent booster dose were excluded from analyses (41). In addition, participants who had received only 1 previous COVID-19 vaccine dose were excluded from analyses (50), because those who did not complete a primary series (or received a 1-dose primary vaccine) might not be eligible to receive a bivalent booster dose or might have different reasons for receiving booster doses. One participant reported both having received 1 dose and also being unsure about their bivalent booster vaccination status; thus, a total of 90 participants were excluded.

** Participants reported all previous SARS-CoV-2 infections for which they had received a positive test result (rapid test or reverse transcription–polymerase chain reaction test).

The 396 participants who had received the bivalent booster dose selected a median of five reasons for getting it.*** The most common reasons were to protect oneself (90.7%), prevent severe disease (80.6%), and protect others (75.0%) (Figure); these top reasons were consistent among age groups.

**FIGURE:**
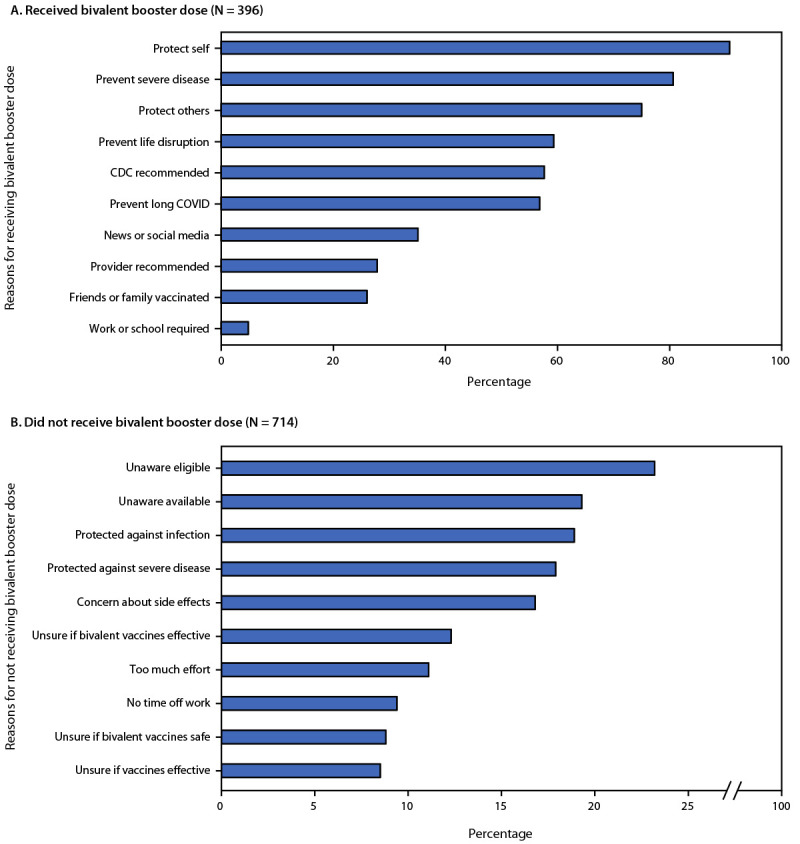
Reasons for receiving (A) or not receiving (B) a bivalent COVID-19 booster dose, among persons who did and did not receive it — United States, November–December 2022

The 714 participants who had not received the bivalent booster dose selected a median of one reason for not receiving it (Figure).^†††^ Reasons for not receiving the bivalent booster dose differed among age groups (Supplementary Figure, https://stacks.cdc.gov/view/cdc/123508). Among adults aged 18–39 years, the most commonly reported reasons for not receiving the bivalent booster dose were being unaware that they were eligible (29.8%), being unaware that updated booster doses were available (23.5%), or believing they still had strong protection against infection (18.4%). The most commonly reported reasons among adults aged 40–59 years were being unaware that they were eligible (22.1%) or believing they still had strong protection against severe disease (21.3%) or infection (18.5%). Among adults aged ≥60 years, the most commonly reported reasons were believing they still had strong protection against infection (20.2%), concern about side effects (17.5%), or being unsure whether the bivalent booster dose was effective (16.1%).

Adults aged 40–59 and ≥60 years commonly reported not receiving a booster dose because they believed they were already sufficiently protected against infection or severe disease. However, among 223 participants who cited one or both of these reasons, 160 (71.7%) had not experienced a SARS-CoV-2 infection or received a COVID-19 vaccine dose within the preceding 6 months, and 114 (51.1%) had never been infected.

Reasons for nonvaccination were descriptively grouped into the following three categories: 1) lack of awareness (related to eligibility and availability), 2) perceived immunity (i.e., self-perceived strong protection against infection or severe disease), and 3) concern and uncertainty (related to vaccine effectiveness, safety, and side effects). Reasons within each descriptive category were more often reported together (co-occurrence frequency >15%)^§§§^ (Table 2).

**TABLE 2:** Co-occurrence* of reasons for not receiving the COVID-19 bivalent booster vaccine among participants who cited two or more reasons (N = 251) — United States, November 1–5, 2022

Reasons for not getting bivalent COVID-19 booster dose	Percentage									
	Unaware eligible	Unaware available	Protected against infection^†^	Protected against severe disease^§^	Concern about side effects	Unsure if vaccines are effective	Unsure if bivalent vaccines are effective	Unsure if bivalent vaccines are safe	Too much effort	No time off work
**Unaware eligible**	NA	—^¶^	—	—	—	—	—	—	—	—
**Unaware available**	47.4*	NA	—	—	—	—	—	—	—	—
**Protected against infection^†^**	5.7	9.6	NA	—	—	—	—	—	—	—
**Protected against severe disease^§^**	8.4	6.2	33.1*	NA	—	—	—	—	—	—
**Concern about side effects**	10.3	2.7	14.2	19.4*	NA	—	—	—	—	—
**Unsure if vaccines are effective**	2.5	0.9	9.2	6.2	18.3*	NA	—	—	—	—
**Unsure if bivalent vaccines are effective**	5.0	1.6	13.3	8.4	22.5*	30.2*	NA	—	—	—
**Unsure if bivalent vaccines are safe**	2.3	2.7	9.4	5.8	15.2*	28.9*	37.9*	NA	—	—
**Too much effort**	4.0	4.0	13.5	11.7	9.2	6.3	7.0	4.5	NA	—
**No time off work**	8.8	5.6	3.5	6.0	9.9	4.1	3.2	1.2	12.7	NA

**Abbreviation:** NA = not applicable.

* Pairs of reasons that co-occurred >15% of the time. Co-occurrence was calculated as the percentage of instances in which a participant cited both reasons, given that at least one of the two reasons was cited. An arbitrary threshold of 15% was used to identify notable co-occurrences. Reasons that frequently co-occurred aligned with the descriptive categories described in the main text (e.g., the “unaware” category included the “unaware eligible” and “unaware available” reasons, which had a co-occurrence frequency of 47.4%).

^†^ Full text of reason: “I believe I still have strong protection against COVID-19 infection.”

^§^ Full text of reason: “I believe I still have strong protection against severe illness due to COVID-19.”

^¶^ Dashes indicate cells that are omitted because the values would be redundant with other cells; the matrix of reasons is symmetric on both sides of the diagonal.

After participants who had not received the booster dose (714) selected their reasons, they read a message about vaccine eligibility and availability and then reported their intention to get the booster dose. Overall, more than two thirds (67.8% [484 of 714]) indicated that they planned to get the booster dose (similar across age groups). Among those who had reported being unaware about eligibility or availability, 88.0% (227 of 258) indicated that they planned to get the booster dose.

After 1 month, the 714 participants who had not received the booster dose at the initial evaluation were recontacted; 624 (87.4%) completed the follow-up survey (Table 1). Among 427 (68.4%) participants who planned to receive the booster dose, 122 (28.6%) had done so. In contrast, among 197 participants who did not plan to receive the booster dose, nine (4.6%) had received it. Among the 305 participants who planned to get the booster dose but had not yet done so, 252 (82.6%) still intended to get it, three (1.0%) no longer planned to get it, and 50 (16.4%) were unsure. Recontacted participants who still had not received the booster dose selected reasons again^¶¶¶^; the most common reasons were being too busy (35.6%), forgetting (22.7%), and worrying about side effects (22.7%).****

## Discussion

In this online survey aimed at understanding reasons for low bivalent COVID-19 booster vaccination coverage, the most common reasons for not receiving a bivalent booster dose were lack of awareness of eligibility (23.2%) or availability (19.3%) and perceived existing protection against infection (18.9%), although top reasons differed across age groups. Bivalent booster dose coverage in the U.S. was low when the survey was conducted (12.1% of adults), and currently remains low (18.2% of adults) (*3*). Increasing bivalent booster vaccination coverage will require a multifaceted approach (*4*) to address reasons for nonvaccination.

Lack of awareness about eligibility to receive a booster dose and vaccine availability were among the three most common reasons for not receiving the booster dose among adults aged 18–39 and 40–59 years. After viewing information about current booster vaccination guidelines, most participants who had been unaware of their eligibility or about availability reported planning to get the booster dose. Increased outreach, such as through provider recommendations and trusted messengers (*4*,*5*), is necessary to increase awareness of eligibility criteria and vaccine availability. Increasing awareness is a crucial first step toward increasing coverage; promotion of tools that provide vaccination guidance (such as CDC’s COVID-19 booster tool)^††††^ by public health authorities and trusted messengers might help encourage persons who are unsure about bivalent booster dose recommendations to receive the booster dose.

Other respondents did not receive a booster dose because they believed they were protected against infection or severe disease because of previous vaccination or infection. These reasons were among those most frequently cited by adults aged 40–59 and ≥60 years. Among participants who cited these reasons, nearly three quarters had not experienced a SARS-CoV-2 infection or received a COVID-19 vaccine dose within the preceding 6 months, and more than one half had never been infected. Because of waning of vaccine- and infection-conferred immunity and evolving viral variants (*6*,*7*), these participants likely overestimated their protection. An online intervention has been shown to correct inaccurate estimation of COVID-19 exposure risk (*8*); similar strategies could correct misconceptions about the need for COVID-19 bivalent booster vaccination, such as interactive online tools that provide personalized immunity estimates.

Some participants expressed concern about bivalent booster dose side effects, safety, and effectiveness. These concerns were among the most frequent reasons for not receiving the booster dose among adults aged ≥60 years. Increasing awareness of emerging safety and effectiveness data related to bivalent booster vaccination among providers and public health messengers could help address these concerns (*1*,*2*,*5*).

After 1 month, 29% of participants who had planned to get the bivalent booster dose had received it; 83% of those who had not yet received a booster dose still planned to receive it. Recontacted participants who had not received the booster dose most commonly reported being too busy, forgetting, or worrying about side effects. Reminders from providers and trusted messengers, accommodations (e.g., time off work to recover), and convenient access (e.g., at workplaces, schools, or shopping centers) might motivate persons to act on their intentions (*4*,*5*). Increased awareness of safety data could also address concerns about side effects.

The findings in this report are subject to at least six limitations. First, most users in the Prolific participant pool identified as White (particularly among adults aged ≥60 years and previously COVID-19–vaccinated adults), making it impossible to weight the sample by race or ethnicity to represent the general U.S. population. Second, the survey used a nonprobability sample, and the cumulative response rate cannot be reported because Prolific does not report the number of users who viewed a survey but did not opt-in. Third, inferences are limited to persons who received ≥2 previous COVID-19 vaccine doses. These three limitations constrain the generalizability of the findings as well as inferences about demographic or geographic differences. Previous studies have demonstrated racial and ethnic disparities in COVID-19 booster vaccination (*9*); reasons for nonvaccination might differ among communities because of work, transportation, or language barriers. Fourth, self-reported information is subject to social desirability and recall biases (*10*); participants might have felt pressured to provide socially desirable answers or inaccurately recalled past experiences, which limits the interpretability of the survey responses. Fifth, the survey only assessed booster vaccination intentions at the end of the survey, making it difficult to determine whether providing information about vaccination eligibility or vaccine availability influenced intentions. Finally, because of selection bias, those who had strong opinions about COVID-19 vaccination might have been more likely to participate.

This study identified lack of awareness, perceived immunity, and concern and uncertainty as important reasons underlying low adult bivalent booster vaccination coverage. All eligible adults should receive a bivalent booster dose to protect themselves against SARS-CoV-2 infection and severe disease. To help increase bivalent booster coverage, health care professionals and public health practitioners should use evidence-based strategies to convey information about booster vaccination recommendations and waning immunity, in addition to increasing convenient access to vaccination.

**Summary**

**What is already known about this topic?**

Bivalent COVID-19 booster vaccines increase protection against infection and severe disease. However, few eligible U.S. residents have received a bivalent booster dose, and factors underlying low coverage are unclear.

**What is added by this report?**

An online opt-in survey of 1,200 previously vaccinated U.S. residents found that the most common reasons for not getting a bivalent booster dose were lack of awareness about eligibility or availability and overconfidence in immunity; reasons varied by age group.

**What are the implications for public health practice?**

All eligible adults should receive a bivalent COVID-19 booster vaccine. To help increase bivalent booster dose coverage, health care and public health professionals should use evidence-based strategies to inform persons about booster vaccine recommendations and waning immunity.

## Footnotes

*
https://www.cdc.gov/vaccines/covid-19/clinical-considerations/covid-19-vaccines-us.html

† Prolific is an online survey platform (www.prolific.co/). Participants were paid $1 for a survey that took 3–5 minutes to complete. After 1 month, participants were recontacted for a 1.5-minute follow-up survey that paid $1. Survey creators recruit participants on the basis of previous responses to a demographic survey administered by Prolific; only participants who met the eligibility criteria were able to view the study and opt in.

§ 45 C.F.R. part 46; 21 C.F.R. part 56.

¶ Suggested reasons for receiving the booster dose are as follows: 1) I wanted to protect myself; 2) I wanted to protect others; 3) I wanted to prevent my own severe illness due to COVID-19; 4) I wanted to prevent my own long-term symptoms due to COVID-19 (long COVID); 5) My doctor (or other health care provider) recommended getting the booster dose; 6) I wanted to prevent life disruption due to COVID-19 (e.g., missing work or vacations); 7) CDC recommended getting the booster dose; 8) My friend or family members got the booster dose; 9) I read content in the news or on social media about the booster dose; and 10) My employer or school required it.

** Participants optionally input other text to describe additional reasons (31.6% among those who had already received the bivalent booster dose, and 53.9% among those who had not). However, because many of these responses were not informative (e.g., writing “nothing to add” or providing a reason that was redundant with a reason already selected from the list), these text-entry responses were excluded from analyses.

†† Suggested reasons for not receiving the booster dose are as follows: 1) I believe I still have strong protection against COVID-19 infection; 2) I believe I still have strong protection against severe illness due to COVID-19; 3) I didn’t want to experience vaccine side effects; 4) I couldn’t take time off work to get the vaccine or recover afterwards; 5) I believe the vaccines are not effective anymore; 6) I didn’t know the new booster was available; 7) I didn’t know if I was eligible for the new booster; 8) It’s too much effort to get the booster shot; 9) I don’t know if the new formula is effective; and 10) I don’t know if the new formula is safe.

§§ Participants read a message describing CDC recommendations and eligibility criteria for bivalent booster doses along with a link to an appointment-finding tool (https://www.vaccines.gov/search/). Click-tracking indicated that 5.7% of participants clicked on the link, although this measure might fail to count some clicks (e.g., if a participant copied and pasted the link or used a keyboard shortcut to open it in a new tab or window). Participants might have been unlikely to click the link because of concerns about losing their place in the survey.

¶¶
https://osf.io/t3szm/

*** 6.6% of participants cited one reason, 5.3% cited two reasons, 9.6% cited three reasons, 14.6% cited four reasons, and 63.9% cited five or more reasons.

††† 7.0% did not cite any of the suggested reasons, 61.3% cited one reason, 22.7% cited two reasons, and 15.3% cited three or more reasons.

§§§ Although only one third of participants cited more than one reason, recommendations based on the descriptive categories (lack of awareness, perceived immunity, and concern and uncertainty) apply to all participants who chose one or more of the reasons associated with each category.

¶¶¶ Suggested reasons for not receiving the booster dose in the follow-up study are as follows (listed in order of prevalence): 1) I’ve been too busy to get the booster dose; 2) I didn’t want to experience vaccine side effects; 3) I intended to get the booster dose, but I forgot to make an appointment; 4) I believe I still have strong protection against COVID-19 infection; 5) I believe I still have strong protection against severe illness due to COVID-19; 6) I don’t know if the new formula is effective; 7) I don’t know if the new formula is safe; 8) I had COVID-19 recently, so I think I should wait before getting a booster dose; 9) I believe the vaccines are not effective anymore; 10) I couldn’t take time off work to get the vaccine or recover afterwards; 11) I am still not sure if I am eligible to receive a booster dose; and 12) I am not sure if vaccine doses (or the specific brand I prefer) are available near me.

**** In the follow-up survey, among the subset of participants (305) who were unsure or no longer planned to get the booster dose, the top reasons for not getting it were being too busy (37.7%), concern about side effects (34.0%), and strong perceived protection against infection (24.5%) and severe disease (24.5%).

††††
https://www.cdc.gov/coronavirus/2019-ncov/vaccines/stay-up-to-date.html
